# Strain-specific features of *Pleurotus ostreatus* growth in vitro and some of its biological activities

**DOI:** 10.1186/s12896-024-00834-9

**Published:** 2024-02-08

**Authors:** Tetiana Krupodorova, Victor Barshteyn, Victoria Tsygankova, Mustafa Sevindik, Yaroslav Blume

**Affiliations:** 1 Department of Plant Food Products and Biofortification, Institute of Food Biotechnology and Genomics of the National Academy of Sciences of Ukraine, Baidy-Vyshnevetskoho Str. 2a, Kyiv, 04123 Ukraine; 2 Department of Chemistry of Bioactive Nitrogen-Containing Heterocyclic Bases, V.P. Kukhar Institute of Bioorganic Chemistry and Petrochemistry, National Academy of Sciences of Ukraine, Academician Kukhar Str. 1, Kyiv, 02094 Ukraine; 3 Department of Food Processing, Bahçe Vocational School, Osmaniye Korkut Ata University, İslam Ali Farsakoğlu Cad No:66, 80000 Bahçe/Osmaniye, Turkey; 4 Department of Genomics and Molecular Biotechnology, Institute of Food Biotechnology and Genomics of the National Academy of Sciences of Ukraine, Baidy-Vyshnevetskoho Str. 2a, 04123 Kyiv, Ukraine

**Keywords:** Oyster, Mycelia, Growth stimulation, Wastes, Antimicrobial, DPPH

## Abstract

**Background:**

The production of *Pleurotus ostreatus* mycelium as a promising object for use in food and other industries is hampered by a lack of information about the strain-specificity of this fungus mycelium growth and its acquisition of various biological activities. Therefore, this research aimed to investigate mycelial growth of different *P. ostreatus* strains on varies solid and liquid media as well as to evaluate strains antagonistic, antibacterial, antiradical scavenging activities, and total phenolic content.

**Results:**

Potato Dextrose Agar medium was suitable for all strains except *P. ostreatus* strain 2460. The best growth rate of *P. ostreatus* 2462 strain on solid culture media was 15.0 ± 0.8 mm/day, and mycelia best growth on liquid culture media—36.5 ± 0.2 g/l. *P. ostreatus* strains 551 and 1685 were more susceptible to positive effect of plant growth regulators Ivin, Methyur and Kamethur. Using of nutrient media based on combination of natural waste (amaranth flour cake and wheat germ, wheat bran, broken vermicelli and crumbs) has been increased the yield of *P. ostreatus* strains mycelium by 2.2–2.9 times compared to the control. All used *P. ostreatus* strains displayed strong antagonistic activity in co-cultivation with *Aspergillus niger*, *Candida albicans*, *Issatchenkia orientalis*, *Fusarium poae*, *Microdochium nivale* in dual-culture assay. *P. ostreatus* 2462 EtOAc mycelial extract good inhibited growth of *Escherichia coli* (17.0 ± 0.9 mm) while *P. ostreatus* 2460 suppressed *Staphylococcus aureus* growth (21.5 ± 0.5 mm) by agar well diffusion method. The highest radical scavenging effect displayed both mycelial extracts (EtOH and EtOAc) of *P. ostreatus* 1685 (61 and 56%) by DPPH assay as well as high phenolic content (7.17 and 6.73 mg GAE/g) by the Folin-Ciocalteu’s method. The maximal total phenol content (7.52 mg GAE/g) demonstrated of *P. ostreatus* 2461 EtOH extract.

**Conclusions:**

It is found that the growth, antibacterial, antiradical scavenging activity as well as total phenolic content were dependent on studied *P. ostreatus* strains in contrast to antagonistic activity. The proposed culture mediums of natural waste could be an alternative to commercial mediums for the production mycelial biomass of *P. ostreatus* strains.

## Introduction

*Pleurotus ostreatus* (oyster mushroom) is a wood-destroying fungus-saprotroph (xylotroph), widespread in the temperate zone, popular culinary mushroom, the second most important commercially cultivated mushroom in the world. *P*. *ostreatus* fruiting bodies, active mycelial biomass as well as culture broth are also a bountiful source of bioactive compounds with useful therapeutic properties as antidiabetic, antimicrobial, antitumor, antiviral, hypocholesterolemic, ımmunomodulatory, prebiotic, antioxidant, hypotensive and many more activities [1, 2]. A diet based on *P. ostreatus* mycelium can support a healthy gut microbiome and potentially reduce the need for antibiotics [3]. In addition, *P. ostreatus* products attracted the attention of scientists around the world due to their natural original, minimum side effects and positive impact on human health [4]. The mycelium of *P. ostreatus* has been effectively used to develop an easily reproducible and eco-friendly method for the biosynthesis of cadmium sulphide quantum dots that may provide unique benefits in diagnostic applications [5]. The useful application of mycosynthesis of nano-nutrients (nano-Ag, nano-TiO_2_ and nano-ZnO) by cultivation of *Pleurotus* sp. has been reviewed [6]. In recent decades, also *P. ostreatus* has been one of the most common species for creating bio-composite materials based on fungal mycelium in modern environmentally friendly technologies [7] and for the bioconversion of industrial wastes [8, 9]. All of the above suggests that the production of *P. ostreatus* mycelium as a promising new safe, healthy mycological product and its use in various industries is becoming increasingly widespread. In this regard, it is important to increase the yield of the resulting mycelium and study its useful biological activity. In addition, the assessment of mycelial mass growth rate on solid and liquid media is considered the initial screening stage for spawning quality assessment. The success of fungi cultivation depends on various factors, but the most important factor is the culture medium, as it supplies the necessary nutrients for the mycelium growth. However, slow growth rates are usually one of the main problems that negatively affects the final cost of production and, thus, still arouses a deep interest among many researchers of *P. ostreatus* growth rates study [10, 11]. Also, the bioconversion of low-value natural waste into a valuable end product – *P. ostreatus* mycelium, has become a key research priority in various studies [10, 12]. Another important aspect of fungi cultivation that should definitely be taken into account is the presence of strain-specific features of fungi growth and synthesis of therapeutically important metabolites [10–14]. A comprehensive, ideally integrated, study of the variations in cultural, morphological, physiological, ecological, and genetic properties of *P. ostreatus* strains will contribute to the creation of a suitable base for high quality selection of strains, and their subsequent successful implementation and use on a commercial basis. The aim of this research was to investigate mycelial growth of different *P. ostreatus* strains on varies solid and liquid media as well as to evaluate strains antagonistic, antibacterial, antiradical scavenging activities, and total phenolic content.

## Material and methods

### Fungal and bacteria cultures

*Pleurotus ostreatus* (Jacq.) P. Kumm, strains 551 (HK-35, Sylvan, USA), 1685 (USA), 2460 (Palmycel 107, Poland), 2461 (Belarus), 2462 (Ukraine) were kindly provided by the Mushroom Culture Collection (IBK) of the M.G. Kholodny Institute of Botany of the National Academy of Sciences of Ukraine [15].

Fungus *Aspergillus niger* Tiegh. IFBG 134 was kindly obtained from the Collection of Strains of Microorganisms and Plant Lines of the Institute of Food Biotechnology and Genomics of the National Academy of Sciences of Ukraine. Other fungi *Candida albicans* (C.P. Robin) Berkhout, *Issatchenkia orientalis* Kudryavtsev, *Fusarium poae* (Peck) Wollenw., *Microdochium nivale* (Fr.) Samuels & I.C.Hallett were kindly supplied from the Microorganisms culture collection of the M.G. Kholodny Institute of Botany of the National Academy of Sciences of Ukraine.

*Staphylococcus aureus* UCM B-4001 (ATCC 6538P), *Escherichia coli* UCM B-901 (ATCC 6633), *Klebsiella pneumoniae* UCM B-7623 were kindly obtained from the Ukrainian collection of microorganisms (UKM, UCM) Institute of Microbiology and Virology named after D.K. Zabolotny National Academy of Sciences of Ukraine.

Stock cultures of fungi were maintained on beer-wort agar slants and of bacteria on Mueller–Hinton agar slants at 4 ºC.

### Cultivation conditions

Growth determination of *P. ostreatus* strains have been examined on different solid culture media: Beer Wort Agar (WA): liquid beer wort at 8 degrees on the Balling scale for sugar content, 10.0 g agar; Czapek-Dox Agar (CZA, Sigma Aldrich, USA); Malt Extract Agar (MEA, Difco, USA), Potato Dextrose Agar (PDA, Difco, USA), Glucose-Peptone-Yeast Agar (GPYA) composed of (g/L): 25.0 glucose, 3.0 yeast extract, 2.0 peptone, 1.0 K_2_HPO_4_, 1.0 KH_2_PO_4_, 0.25 MgSO_4_·7H_2_O, and 10.0 agar.

Mycelial biomass of *P. ostreatus* strains have been evaluated in liquid media. The basis (60 g per 1 L of distilled water) of liquid medium were different wastes (composition – in accordance with the regulations of the manufacturer): cakes of wheat germ (LLC «Zhitomirbioprodukt», Ukraine) and of amaranth flour after CO_2_-extraction (*Amaranthus hybridus* L. grains were a variety «Ultra» (Mykolaiv Oblast, Ukraine); wheat bran (LLC «Ukrzerno Industrial Supplies», Ukraine), crumb (waste from pasta production: 5% residue on polyamide fabric sieve No. 43 or No. 49/52) and broken vermicelli—threaded pasta that has not passed testing for one or more HACCP parameters (PLC «Macaroni Factory», Ukraine). Waste was used as a monocomponent (60 g per 1 L of distilled water) in a liquid medium and in their combination in a ratio of 1:1. The control refers was liquid Glucose-Peptone-Yeast (GPY) medium.

Synthetic low-molecular-weight heterocyclic compounds represented by pyridine and pyrimidine derivatives, namely have been known as plant growth regulators such as N-oxide-2,6-dimethylpyridine (Ivin), sodium salt of 6-methyl-2-mercapto-4-hydroxypyrimidine (Methyur), potassium salt of 6-methyl-2-mercapto-4-hydroxypyrimidine (Kamethur) were synthesized and kindly obtained from the Department for Chemistry of Bioactive Nitrogen-Containing Heterocyclic Compounds, V.P. Kukhar Institute of Bioorganic Chemistry and Petrochemistry of the NAS of Ukraine. Ivin, Methyur, or Kamethur in concentrations of 10^–6^, 10^–7^, 10^–8^, 10^−9^ M (mol/l) were added to liquid GPY medium at the stage of its preparation.

The prepared medium underwent autoclaving at a temperature of 121 ºC for a duration of 15 min. *P. ostreatus* strains were transferred from preserved cultures onto PDA Petri plates and incubated at a temperature of 26 ± 1 °C in order to cultivate mycelial colonies. The inoculum (one or three mycelial plugs for solid or liquid medium, respectively) was prepared by cutting the mycelial plugs using a sterile borer with a diameter of 8 mm at the stage of active mycelial development. Cultivation on solid and liquid media except study of the impact of chemical compounds was carried out at static cultures (without agitation and in the dark) in flasks for 14 days at 25 ℃ in the dark. The effect of chemical compounds on the growth of *P. ostreatus* strains was determined depending on the cultivation duration (7th, 14th, and 21st- days of cultivation) at the same conditions.

The growth rate (mm/day) was determined on solid media according to the following equation:

$$\mathrm{Growth}\;\mathrm{rate}=\frac{\mathrm{Colony}\;\mathrm{diameter}\;\mathrm{on\ last}\;\mathrm{day}\;(\text{mm})}{\mathrm{Number}\;\mathrm{of}\;\mathrm{daily}\;\mathrm{measurements}\;\mathrm{taken}\;\mathrm{after}\;\mathrm{inoculation}}$$

The mycelial biomass of *P. ostreatus* strains at growth on liquid media was estimated by the absolute dry weight of the mycelium (a.d.w.). Mycelium grown in liquid medium was separated from the medium by filtration throught Whatmanʼs filter paper N 4, washed with deionized distilled water (ddH_2_O) and dried at a temperature of 105 °C to constant weight for mycelial biomass determination.

### Preparation of fungal extracts

The mycelium was first washed with ddH_2_O, dried at 60 °C, and ground in a blender. Then, 1 g of each strain mycelium was added to a 100-mL conical flask, mixed, 10 mL of solvents like 96% ethanol (EtOH), ethyl acetate (EtOAc), were added, and orbital shaking (100 rpm) was performed for 48 h at room temperature. In the next step, the supernatant was collected by centrifugation for 10 min at 4500 rpm. The resulting supernatant was then collected and filtered through a 25 μm pore size filter (Filter Paper Grade 4). The prepared sample was stored at 4 °C for further use.

### Antagonistic activity

The evaluation of fungal competition between the strains under study and micromycete species was conducted by assessing their capacity to inhibit mycelial growth in dual-culture experiments on Petri dishes containing PDA medium. This assessment involved the use of a rating scale that categorized the reactions into three primary types (A, B, C) and four sub-types (C_A1_, C_B1_, C_A2_, and C_B2_). Additionally, the antagonism index (AI) was calculated using the methodology described by Badalyan et al. [16, 17]. The studied micromycetes together with *P. ostreatus* were incubated in a thermostat with forced air circulation in a chamber (TC-80; Medlan, Kyiv, Ukraine) at 26 °C in the dark at 75% relative humidity. The growth of fungi was monitored daily for a month, and compared with mycelia taken from the controls. The controls refer of each fungus growing individually under the same conditions. Any morphological changes in interacting colonies were monitored. Three replicates were prepared for each pairing.

### Antibacterial assay

The antibacterial activity of the extracts was investigated using the agar well diffusion method. The bacterial strains were cultured in Mueller Hinton Broth medium (MHB, Merck) at a temperature of 37 °C for a duration of 24 h. Bacterial suspensions with a concentration of 10^6^ colony-forming units per milliliter (CFU/mL) for each bacterium were produced and then cultured on Mueller–Hinton Agar. The agar was prepared using paper discs of 7 mm in diameter, onto which 10 µl of extract sample was applied. The determination of inhibitory zones was conducted during a 24-h incubation period at a temperature of 37 °C.

### Radical scavenging activity

The measurement of free radical scavenging activities of fungal extracts was conducted using 2,2-diphenyl-1-picryl-hydrazyl (DPPH) according to the methodology reported in a previous study [18]. A volume of 100 µL of methanol extract at different concentrations was combined with 2900 µL of a DPPH solution (120 µM) in methanol. The mixture was then incubated in a dark environment at a temperature of 37 °C for a duration of 30 min. The control in this experiment consisted of a mixture of methanol and DPPH, without the inclusion of fungal extract. The measurement of absorbance was conducted at a wavelength of 517 nm, and the inhibition of DPPH was determined by calculating the percentage of radical scavenging activity for each fungal extract. This calculation was performed using the provided equation.

$$\mathrm{Percentage}\;\mathrm{of}\;\mathrm{radical}\;\mathrm{scavenging}\;\mathrm{activity}\hspace{0.17em}=\lbrack(\mathrm{Acontrol}-\mathrm{Asample})/\text{Acontrol}\rbrack\times\hspace{0.17em}100$$

where A is the absorbance reading.

### Determination of the total phenolic content (TPC)

The quantification of total phenolic content was conducted with the Folin–Ciocalteu reagent [19]. A 1 ml sample was dissolved in a solution containing 1.5 ml of distilled water and 0.5 ml of Folin–Ciocalteu's reagent. Following a duration of one minute, a volume of one milliliter of a sodium carbonate solution with a concentration of 20% was introduced. The final solution underwent three rounds of agitation and was thereafter subjected to a 2-h incubation period in a lightless environment at a temperature of 25 °C. The measurement of absorbance was conducted at a wavelength of 760 nm. The total phenolic content (TPC) was determined by using a calibration curve with gallic acid as the standard. The quantification was represented as milligrams of gallic acid equivalents per gram of dry weight of the extract (mg GAE/g).

## Statistical analysis

The mean results of all studies were generated from three replications, and each replication had at least three trials. The values were expressed using the mean plus or minus the standard deviation. An analysis of variance (ANOVA) was performed to examine the significance of differences across variables. The one-way ANOVA was computed using Statistica 11.5, a software program developed by StatSoft Inc. in the United States. To determine significant differences, the Fischer's least significant difference (LSD) test was used, with a significance level set at *P* < 0.05.

## Results

### Myceial growth on solid and liquid nutrient media

**Fig. 1:**
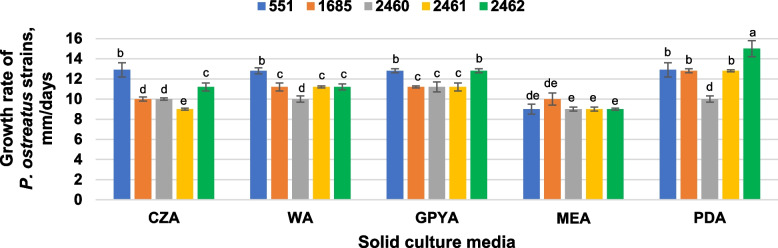
Growth of *Pleurotus ostreatus* strains on solid media: CZA—Czapek-Dox Agar, WA—Worth Agar, GPYA—Glucose-Peptone-Yeast Agar, MEA—Malt Extract Agar, PDA—Potato Dextrose Agar. Mean values (x ± SD, *n* = 3) followed by the same letters are not significantly different on the Fischer`s LSD test (*p* < 0.05)

*P. ostreatus* strains were successfully grown on solid media. The vegetative mycelial growth rate of *P. ostreatus* strains ranged from 9.0 ± 0.1 to 15.0 ± 0.8 mm/day depending on the nutrient medium and strain used (Fig. 1). PDA medium was suitable for all strains except *P. ostreatus* 2460. For this strain GPYA was more appropriated than others. Slowest mycelial growth of all used strains was observed on MEA. The maximum growth rate was established for *P. ostreatus* strain 2462 (15.0 ± 0.8 mm/day on PDA). Of note is *P. ostreatus* strain 551 with a fairly broad adaptation to solid media due to its ability to grow on four of the five agar media at the same growth rate. Also, for other cultures no significant difference was detected between growth efficiencies of some media: WA and GPYA, as well as CZA and MEA for *P. ostreatus* strains 1685 and 2461; CZA, WA, and PDA for strain 2460; CZA and WA in case of 2462 growth. Since GPYA medium over other better supported the growth for all used strains, it was involved as control medium in the next stages of the study.

**Fig. 2:**
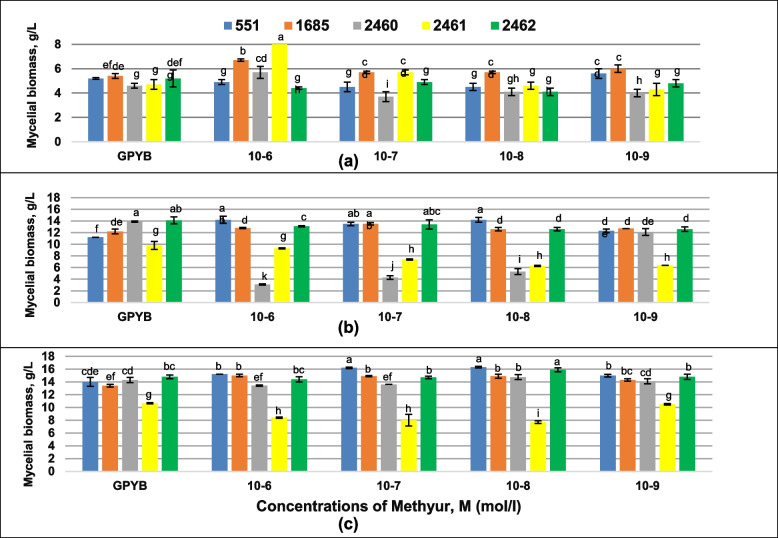
Effect of Methyur on mycelial biomass growth of *Pleurotus ostreatus* strains after 7 **a** 14 **b** and 21 **c** days of cultivation. Mean values (x ± SD, *n* = 3) followed by the same letters are not significantly different on the Fischer`s LSD test (*p* < 0.05)

**Fig. 3:**
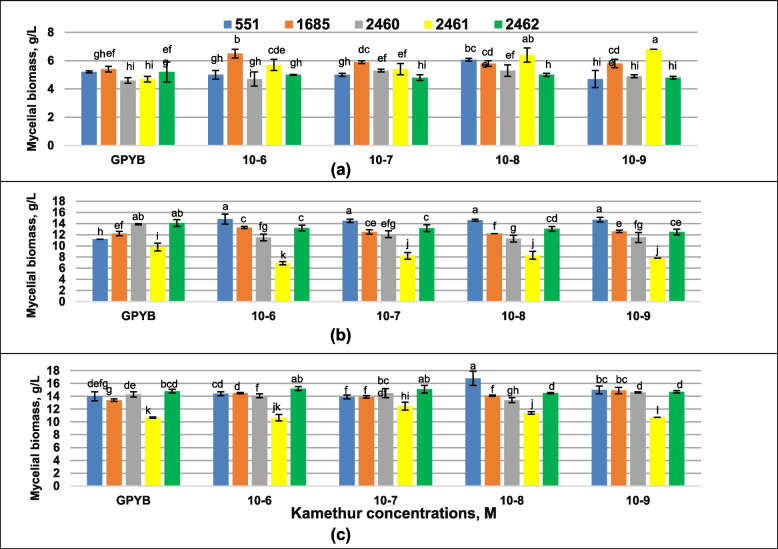
Effect of Kamethur on mycelial biomass growth of *Pleurotus ostreatus* strains after 7 **a** 14 **b** and 21 **c** days of cultivation. Mean values (x ± SD, *n* = 3) followed by the same letters are not significantly different on the Fischer`s LSD test (*p* < 0.05)

**Fig. 4:**
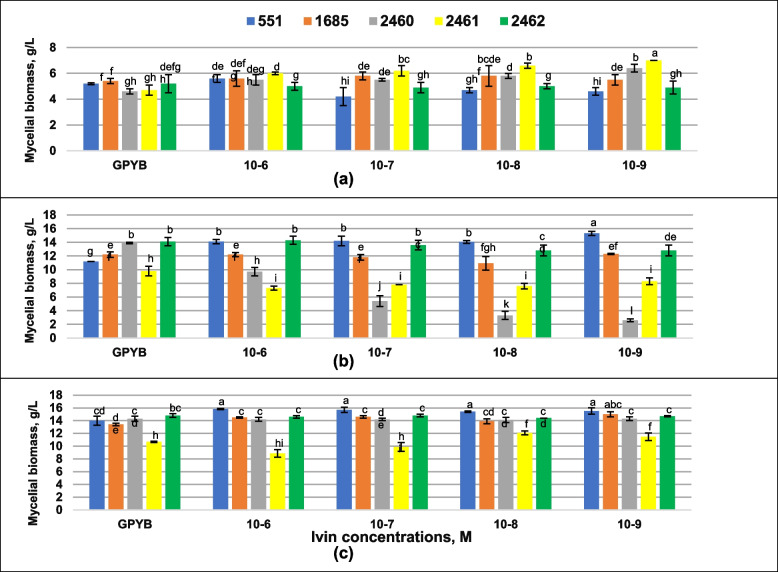
Effect of Ivin on mycelial biomass growth of *Pleurotus ostreatus* strains after 7 **a** 14 **b** and 21 **c** days of cultivation. Mean values (x ± SD, *n* = 3) followed by the same letters are not significantly different on the Fischer`s LSD test (*p* < 0.05)

Two strategies were used to study the stimulation of biomass yield (addition of synthetic plant growth regulators and use of agro-industrial waste as a nutrient medium). The *P. ostreatus* strains grew on synthetic plant growth regulators Methyur, Kamethur and Ivin under the first strategy. The results obtained showed different effects of these chemical compounds on the yield of *P. ostreatus* mycelium. The effect (positive, negative or neutral) of synthetic plant growth regulators on the biomass yield of cultures varied depending on the strains of *P. ostreatus*, the duration of cultivation, and the concentration of the respective chemical compound (Figs. 2, 3 and 4). The addition of Methyur in all tested concentrations ranged from 10^–6^ to 10^−9^ M led to an increase in the biomass yield of *P. ostreatus* strain 1685; the addition of Methyur at a concentration of 10^–6^ M led to an increase in the biomass yield of *P. ostreatus* strain 2460; the addition of Methyur at concentrations of 10^–6^ and 10^–7^ M led to an increase in the biomass yield of *P. ostreatus* strain 2461, as well as the addition of Methyur at a concentration of 10^–9^ M led to an increase in the biomass yield of *P. ostreatus* strain 551 after cultivation for 7 days (Fig. 2a).

All tested concentrations of Methyur supported better growth of *P. ostreatus* strains 551 and 1685 after cultivation for 14th (Fig. 2b) and 21st day (Fig. 2c). Also, longer cultivation (21st day) with addition of Methyur only in concentration 10^–8^ M had positive impact on growth of *P. ostreatus* strain 2462 (Fig. 2c). Some concentrations of Methyur, such as 10^–7^, 10^–8^, 10^–9^ M, had a negative effect on the growth of *P. ostreatus* strain 2460 on the 7th day (Fig. 2a), and the concentrations of Methyur, such as 10^–6^ and 10^–7^ M had a negative effect on the growth of *P. ostreatus* strain 2460 after 21st day of growth (Fig. 2c).

All tested concentrations of Methyur reduced the growth of mycelium of the strains 2460, 2461, 2462 on the 14th day of cultivation (Fig. 2b). In other cases (strain, Methyur concentration and duration of cultivation), the neutral effect of Methyur on growth of *P. ostreatus* strains was found.

The addition of Kamethur at all concentrations ranged from 10^–6^ to 10^−9^ M supported growth of *P. ostreatus* strains 1685 and 2461; the addition of Kamethur at a concentration of 10^–7^ M stimulated growth of the *P. ostreatus* strain 2460, the addition of Kamethur at a concentration of 10^–8^ M stimulated growth of *P. ostreatus* strains 551, 2460, 2461, respectively, on the 7th day of growth (Fig. 3a). Other concentrations led to neutral effect on cultures after 1 week of growth. All tested concentrations of Kamethur showed the positive effect only on growth of *P. ostreatus* strain 551 and revealed the negative effect on growth of *P. ostreatus* strains 2460, 2461 and neutral effect on growth of *P. ostreatus* strains 1685 and 2462 after 14 days of experiment (Fig. 3b). Longer cultivation (21 day) leads to the growth of *P. ostreatus* strain 1685 when using Kamethur at all tested concentrations, *P. ostreatus* strain 2462—when using Kamethur at a concentration of 10^–7^ M, *P. ostreatus* strain 551 when using Kamethur at a concentration of 10^–8^ M, while in other cases a neutral effect of Kamethur was found (Fig. 3c).

The addition of Ivin at all studied concentrations led to an increase in the biomass of the strains 2460 and 2461, and a neutral effect on growth of *P. ostreatus* strains 1685 and 2462 was observed after 7 days of cultivation. The biomass yield of *P. ostreatus* strain 551 increased with Ivin addition of 10^–6^ M. Decrease in the concentration of Ivin from 10^–7^ to 10^–9^ M had a negative effect on the growth of *P. ostreatus* strain 551 after first week of experiment (Fig. 4a). Cultivation after two weeks showed that all tested concentrations of Ivin led to a positive effect on growth of *P. ostreatus* strain 551, a neutral effect on growth of *P. ostreatus* strains 1685 and 2462, and a decrease in growth of *P. ostreatus* strains 2460, and 2461 (Fig. 4b). Longer cultivation (21 days) led to an increase in the biomass yield of *P. ostreatus* strain 2461 when using Ivin at the concentrations of 10^–8^ and 10^–9^ M, as well as increase in the biomass yield of *P. ostreatus* strains 551, 1685 at all tested concentrations, and had a neutral effect on *P. ostreatus* strains 2460, 2462. The use of Ivin at a concentration of 10^–6^ and 10^–7^ M led to a decrease in the growth of *P. ostreatus* strain 2461 over three weeks of the experiment (Fig. 4c).

**Fig. 5:**
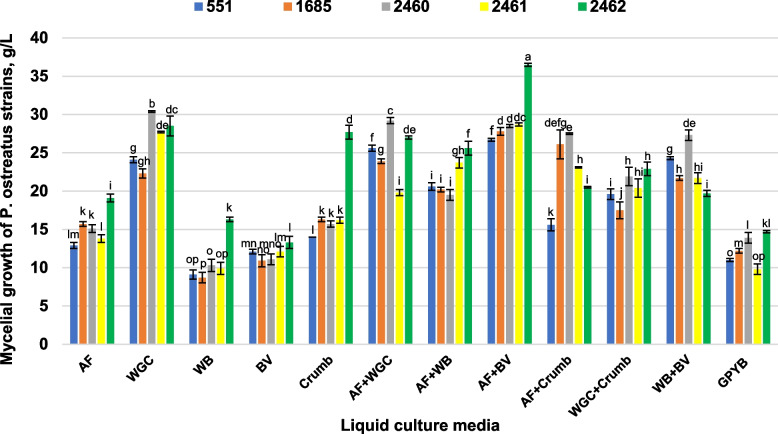
Effect of liquid media on mycelial biomass growth of *P. ostreatus* strains. Liquid media: AF- amaranth flour; WGC- wheat germ cake; WB- wheat bran; BV- broken vermicelli; Crumb as waste from pasta production; CPYB- glucose-peptone-yeast broth (Control). Mean values (x ± SD, *n* = 3) followed by the same letters are not significantly different on the Fischer`s LSD test (*p* < 0.05)

Successful biomass growth of all *P. ostreatus* strains was obtained using the second strategy. Mycelial yield of the tested cultures ranged from 8.7 ± 0.7 to 36.5 ± 0.2 g/l, depending on the strain and test medium (Fig. 5). A significant effect of 9 liquid natural media based on waste on the mycelial growth of all *P. ostreatus* strains was revealed. Among the one-component bases, wheat germ cake followed by the addition of crumbs and amaranth flour better supported the growth of all studied *P. ostreatus* strains.

Other mono-substrates did not have a positive effect on the growth of all strains, however, their use in combination with other substrates contributed to the growth-stimulating effect. In general, the combination of used natural bases in the culture medium significantly increased the biomass yield. The best result was obtained by combining amaranth flour and broken vermicelli waste. The maximum amount of biomass was produced by strain 2462 on most of the media used. Strain 2460 also well assimilated the studied media (Fig. 5).

### Biological activities

**Fig. 6:**
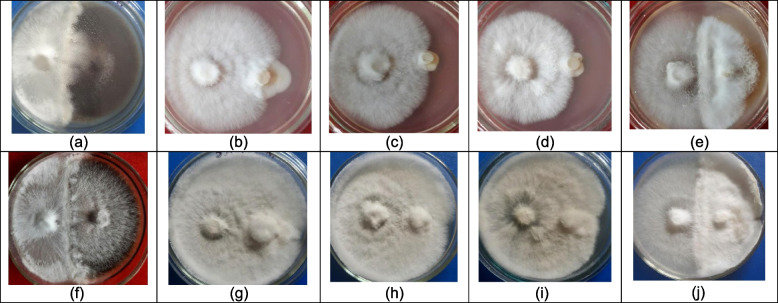
Interspecific interactions between mycelium of *P. ostreatus* strains (left) and studied fungi (right): *P. ostreatus* 551 and *A. niger* on 7 **a** and 14 days of co-cultivation **f** *P. ostreatus* 551 and *M. nivale* on 7 **b** and 14 days of co-cultivation **g** *P. ostreatus* 1685 and *C. albicans* on 7 **c** and 14 days of co-cultivation **h** *P. ostreatus* 2460 and *I. orientalis* on 7 **d** and 14 days of co-cultivation **i** *P. ostreatus* 2461 and *F. poae* on 7 **e** and 14 days of co-cultivation **j**

For all studied strains of *P. ostreatus,* antagonistic activity was noted against the used micromycetes *A. niger*, *C. albicans*, *I. orientalis*, *F. poae* and *M. nivale.* Co-cultivation of the studied *P. ostreatus* strains based on their relative combative ability resulted in the one reaction (subtype of interaction C_A2_)—complete replacement after initial deadlock on contact (Fig. 6). All *P. ostreatus* strains revealed strong antagonistic activity with high level of antagonistic index (total AI = 22.5). Morphology of contact fungal colonies was unchanged.

Only some extracts of *P. ostreatus* strains possessed antibacterial action. The EtOAc extract of *P. ostreatus* 2462 showed strong antibacterial activity against *S. aureus*, and in the case of *P. ostreatus* 2460—against *E. coli*. The zones of growth inhibition were 21.5 ± 0.5 mm and 17.0 ± 0.9 mm, respectively. Our studies have shown that other extracts of *P. ostreatus* strains did not exhibit any potential antimicrobial activity against *E. coli*, *S. aureus* and *K. pneumoniae*.

**Fig. 7:**
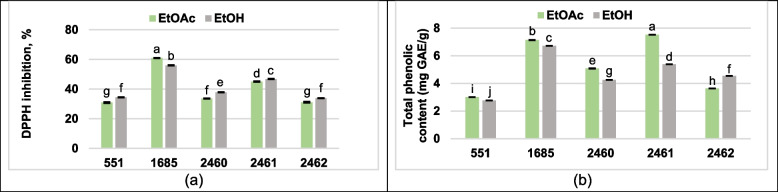
Scavenging activity of extracts from the *P. ostreatus* strains against DPPH **a** and total phenolic content **b** Mean values (x ± SD, *n* = 3) followed by the same letters are not significantly different on the Fischer`s LSD test (*p* < 0.05)

The evaluation of the scavenging activity of extracts by the DPPH assay was expressed as percentage inhibition as presented in the graph below (Fig. 7a). The percentage DPPH inhibition from 30.9 to 61 depended on the studied *P. ostreatus* strain and on the solvents used for extract preparation of mycelium. However, significant differences in the DPPH inhibition were not found in comparison between *P. ostreatus* strains 551 and 2462. These strains of *P. ostreatus* were exhibited the weakest radical scavenging activity. Slight better DPPH inhibition was established for EtOH extracts except *P. ostreatus* strain 1685. At the same time, among the studied strains the most potent activity was found for both extracts of *P. ostreatus* strain 1685 (61 and 56%).

The TPC of all fungal extracts under study was found in ranging from 2.76 to 7.52 mg of GAE/g as Gallic Acid equivalents (Fig. 7b). The maximum amount of TPC was recorded with the EtOAc extract of *P. ostreatus* strain 2461 (7.52 mg of GAE/g) followed by *P. osteratus* strain 1685 and it EtOH extract with 7.17 and 6.73 mg of GAE/g, respectively. The TPC level statistical depended on the studied *P. ostreatus* strain as well as on the solvents used for extract preparation of mycelium. EtOAc solvent, with the exception of *P. ostreatus* strain 2462, was more suitable for TPC isolation than EtOH solvent.

## Discussion

### Myceial growth on solid and liquid nutrient media

*P. ostreatus* is the object of intensive research due to its unique attributes, which open up broad prospects for its application in biotechnology and various sectors of modern industry [6]. Finding the optimal growing medium for successful mushroom growth is one of the first steps to ensure that they can be used efficiently and cost-effectively in the future. The present comparative studies on the growth of *P. ostreatus* strains on five agar media revealed that the cultures generally grew fast and differently on all the solid media tested. Differences in mycelial growth of *P. ostreatus* strains may be related to their availability of appropriate nutrients, primarily sources of carbon, nitrogen, also vitamins, macro- and microelements. This study allowed us to establish favorable and alternative solid agar media for the growth of each strain. The studied strains, according to the degree of greater adaptive ability to the same grow on the used solid nutrient media, can be presented in the following order: 2462 > 2460 > 2461 = 1685 > 551. In general, GPYA medium supported the growth of all used strains better than others. This result confirmed previous evidence of the ability of glucose to be rapidly catabolized by fungi for easy cellular energy production [20]. However, the highest growth rate of most strains was observed on PDA medium. The best growth rate was found for the strain *P. ostreatus* 2462 (15.0 ± 0.8 mm/day on PDA). It was previously found that this medium is also best suited for mycelial growth of other strains of *P. ostreatus* under different study [14, 21–23]. In contrast to our observations, the best mycelial growth of *P. ostreatus* was observed in MEA [24, 25].

One of the most pressing tasks of biotechnology is to find ways to increase the fungal yield when they are grown in artificial conditions. To solve this problem, culture media are saturated with various additives of natural or synthetic origin. Use of plant growth regulators is one of tools to augment yield of *P. ostreatus*. Earlier investigators have reported significant role of growth regulators such as gibberellic acid (GA) in the growth and yield performance of *Pleurotus ostreatus* [26–29]. Hong [30] observed supporting the growth of *P. ostreatus* with the addition of GA, kinetin, indole acetic acid (IAA) at doses 10 ppm, 0.1 as well as 0.01 ppm, and 0.1 ppm respectively. Reddy et al. [31] found the best *P. ostreatus* growth using IAA at 5 ppm. Influence of some growth regulators on development of vegetative mycelium of *P. ostreatus* on agar media also has been studied [32–34]. Vinklárková and Sladký [32] reported that the best growth of *P. ostreatus* was observed on media with 100 ppm IAA, 200 ppm GA and 200 ppm of kinetin. Zakolesnyk [33] noted that growth regulators such as Fumar, GA and heteroauxin have positive impact on primordia formation depend of their concentrations, but they not uniquely act on mycelium growth of *P. ostreatus* HK-35 (IBK 551) on agar media with corn broth. Kuznetsova [34] observed a positive effect of Fumar (10^–3^% and 10^–4^%) and Biogumat (10^–2^%) on the radial growth rate of *P. ostreatus* HK-35 (IBK 551) mycelium, while heteroauxin at a concentration 10^–4^% inhibited mycelium in the linear growth phase. These results clearly show that at certain concentrations, plant growth regulators can stimulate the growth of *P. ostreatus*. Recently, synthetic low-molecular-weight heterocyclic compounds represented by pyridine, pyrimidine, pyrazole and oxazole derivatives have been under extensive research due to their high efficiency at low concentrations and lack of toxic effects on plant and animal cells, which ensures environmental safety [35–40]. To the best of our knowledge, in this study we examined the effect of synthetic plant growth regulators Ivin, Methyur and Kamethur on fungal growth is the first time. The results obtained showed positive, negative and neutral effects of these chemical compounds on the yield of *P. ostreatus* mycelium.These data demonstrate that the effect of these chemical compounds was highly dependent on the strain, as well as the duration of cultivation and the concentration of the respective compound used. It should be noted that no difference in the influence of the concentration of the chemical compounds used was found only when *P. ostreatus* cultures were cultivated for two weeks. The studied synthetic compounds can be arranged in the following order (according to the degree of fungal growth stimulation): Kamethur > Ivin > Methyur. A certain chemical similarity between Methyur and Kamethur at all concentration used had the same growth-regulating effect on the 7th day of the experiment for strains 551, 1685 and 2462, on the 14th day of the experiment for strains 551, 2460, 2461, and on the 21st day of the experiment only for strain 1685. In addition, all three chemical compounds used at all concentrations had the same growth-regulating effect on the *P. ostreatus* strains 551, 2460, 2461 when cultivated for two weeks and on the *P. ostreatus* strain 1685 when cultivated for three weeks. Among all studied cultures, *P. ostreatus* strains 551 and 1685 were more receptive to positive assimilation of all investigated compounds used in nanomolar concentrations. However, the growth-stimulating activity of the synthetic plant growth regulators Ivin, Methyur and Kamethur was previously established for various crops [37–39, 41–43]. In general, concentrations of 10^–5^, 10^–6^ and 10^−7^ M of these synthetic plant growth regulators showed the highest growth-regulating effect [37–39, 41–43].

Despite a certain increase in the stimulating effect under the influence of synthetic plant growth regulators, the second strategy used to increase the biomass yield turned out to be more effective. According to the results obtained, the use of nutrient media based on a combination of natural waste makes it possible to increase the yield of mycelium of *P. ostreatus* strains by 2.2–2.9 times compared to the control. It is well known that fungi need carbon, nitrogen, and inorganic compounds as a source of nutrition. The medium prepared on the basis of natural waste allows fungal cultures to receive the necessary nutrients in an accessible form, such as crude protein, crude fiber, nitrogen, vitamins, minerals, which can support their growth. The bioavailability of nutrients in the medium is determined by the unique physiological and biological characteristics (above all, ability to secrete specific enzymes) of the cultures and has a strain-specific dependence. It should be noted that strain 2462, which had the maximum growth on solid PDA medium, also showed the best results of biomass production when growing on most of the studied liquid media. In general, the studied wastes can be a good alternative basis for the production of mycelium as a seed (spawn) and biomass, and therefore as well as an effective additional component to substrates for the production of fruiting bodies. Since substrates with a high biomass return, which can pose environmental problems, can be a good alternative for the cultivation of *P. ostreatus*. In this regard, the potential use of such studied waste as an alternative medium for mycelial production deserves to be explored also in industry conditions and confirmed through further research in this field. Successful bioconversion of amaranth flour into mycelial biomass was recorded in our previously studies [8, 44, 45]. Meanwhile, according to our early observations, mycelium of higher fungi obtained using amaranth flour had antibacterial [44], antiviral [46], anticancer [47] and wound healing activities [48]. Our current study also demonstrated for the first time positive effect of amaranth flour waste in combination with other wastes studied on the increase in the mycelial mass production of used *P. ostreatus* strains. The possibility and effectiveness of using natural waste (as a monobase or as a component of a liquid culture medium) to increase the yield of *P. ostreatus* mycelium has been shown in some studies [49, 50].

Taking into account the data obtained in this work, a promising issue of our further research is the study of the combined effect of synthetic plant growth regulators Ivin, Methyur and Kamethur and natural waste to increase the biomass yield of *P. ostreatus* strains.

### Biological activities

In addition to its high food and industrial value, *P. ostreatus* continues to play a significant role in medicine and pharmacy. Infectious diseases leaded to fungi and bacteria represent an important cause of morbidity and mortality among the general population. The use of crude extracts of *Pleurotus* species and phytochemicals, of known antimicrobial properties, can be of great significance in the therapeutic treatments. Although a certain *P. ostreatus* strain have been early studied with respect to their phytochemical constitutes and antimicrobial potential, their physiological interactions between microbes remain poorly understood. Considering that not only species but also strains of fungi can differ significantly in their properties, the search for fungi antimycotic properties is the first targeted stage of selection of promising fungicidal agents. Current study focuses on the mycelial interactions of *P. ostreatus* strains with 5 fungi, including filamentous and yeast. We selected pathogenic and opportunistic micromycetes as the object of our research. *A. niger, C. albicans*, *I. orientalis*, and *F. poae* cause diseases in humans and animals, including aspergillosis, candidiasis, toxicosis. *A. niger, F. poae*, and *M. nivale*, are widespread pathogens of important agricultural feedstuffs as well as also associated with spoilage of various food products, resulting in significant economic losses. Simultaneously these filamentous fungi are often opportunistic human and animal pathogens [51–53]. Despite the different ecological and trophic requirements of the selected micromycetes, strong antagonistic activity against *A. niger*, *C. albicans*, *I. orientalis*, *F. poae* and *M. nivale* was recorded for all the used *P. ostreatus* strains. The absence of variability in the types of reactions, the level of their visualization, as well as morphological changes in the contacting colonies and the presence of the same level of antagonistic index indicate that fungal-fungal interactions were independent of the strain. The results of the study may not reflect true ecological relationships due to the possible influence of abiotic and biotic factors in the environment, but this study offers a preliminary but rapid way to study and predict how the tested strains of *P. ostreatus* will interact with pathogenic fungi. Understanding the response of microbial communities and possible changes in the habitat can provide valuable information on microbial competition of fungi. To the best of our knowledge the antagonistic activity of *P. ostreatus* against *F. poae* and *M. nivale* was studied in this study for the first time in dual culture. Our results are consistent with the data on the successful antagonistic activity and the ability of *P. ostreatus* mycelium to replace the mycelium of *Pythium* spp. [54], *Bipolaris sorokiniana*, *Fusarium culmorum*, *Gaeumannomyces graminis* var. *tritici*, and *Rhizoctonia cerealis* [16], *Clonostachys rosea* and *Trichoderma pseudokoningii* [17], *Issatchenkia orientalis* and *Candida albicans* strains [55], *Aspergillus niger* and *Penicillium polonicum* [56] under similar co-culture conditions. Moreover, further study of the effects on living organisms will be important to deepen our understanding of these compounds and their potential therapeutic use.

Antimicrobial resistance is a serious global threat that is causing increasing concern for human, animal and environmental health. The growing prevalence of multidrug-resistant strains such as *Escherichia coli*, *Klebsiella pneumoniae* and *Staphylococcus aureus* in the world led us to the selection of these bacteria for our study. To solve the problems of resistance to antibacterial, metabolites from natural object, including higher mushrooms, are increasingly attracting the attention of researchers. In addition, often such products do not have side effects, unlike chemical products. The preliminary screening for antimicrobial activity in this study showed that only the EtOAc extract of two *P. ostreatus* strains, namely 2460 and 2462, showed good growth inhibition of *Staphylococcus aureus* ATCC 6538P and *Escherichia coli* ATCC 6633, respectively. The obtained results in agreement with the findings to those performed by Vamanu [57] regarding the ethanolic mycelium extracts from *P. ostreatus* strain PQMZ91109 also possesses antibacterial effect against *E. coli* CBAB 2, *S. aureus* ATCC 6588. Similarly, it was previously observed by Younis et al. [58] that an aqueous extract of *P. ostreatus* mycelium inhibited the growth of *S. aureus* RCMB (B001001”3”), while it had no effect against *E. coli* RCMB (B004001”4”), but led to inhibition of *Klebsiella pneumoniae* RCMB growth (B008001”2”). In addition, our results are in line with the report of El-Rahman et al. [59], who observed inhibition of the growth of *S. aureus* ATCC 6588 as well as *Escherichia coli* CBAB_2_ when applying *P. ostreatus* extract prepared using a solvent mixture consisting of methanol: glycerol: water (1:1:1 v/v). Also, hot-water mycelial extract from *Pleurotus* sp. strain CCEBI-3024 led to the autolytic enzyme system of the microbial cell of *S. aureus* ATCC 25953 [60]. However, our results are contrary to those performed by Owaid et al. [61] regarding to absent of antibacterial action against *E. coli* ATCC 25922 and *S. aureus* HIP10267. The native mycelium of *P. ostreatus* strain 551 in our previous study [46] was also inactive against *E. coli* 06 and *S. aureus* 209.

The analysis of DPPH free radical scavenging activity is one of the most common methods for determining antioxidant capacity, can accept an electron or hydrogen atom to become a stable diamagnetic molecule. Our result shows that all extracts of *P. ostreatus* strains have DPPH radical scavenging activity, but the percentage of inhibition depends on the strain of *P. ostreatus* as well as on the solvents used. The highest radical scavenging effect of *P. ostreatus* 1685 (61%) was quite close to the results of previous studies with hot water extracts of *P. ostreatus* [62] and some results obtained with ethanol extracts of mycelium of *P. ostreatus* strain PQMZ91109 [57], but lower than the results obtained in other studies using hot water extract. However, all extracts of studied *P. ostreatus* strains possessed more antioxidant activity compare to observation performed by El-Rahman et al. [59] mentioned influence of mycelial extract prepared with solvents mixture (methanol: glycerol: water).

The antioxidant capacity is a way of reflecting the effect of reducing compounds in the fungal extract. The observed free radical scavenging activity can be attributed to the presence of phytochemicals, in particular, phenolic compounds. TPC in both mycelial extracts of *P. ostreatus* strain 1685 and EtOH extract of *P. ostreatus* strain 2461 was similar to reported amounts in other *P. ostreatus* extract prepared from mycelium [57, 61]. In addition, all the extracts of the studied *P. ostreatus* strains had a higher TPC compared to the results of El-Rahman et al. [59] when exposed to mycelial extract prepared with a mixture of solvents (methanol: glycerol: water). It is well known that each solvent has its own specific selectivity for the extraction of different compounds that contribute to the overall antioxidant activity. However, it is important to note that even if the EtOAc extract the highest amount of TPC from *P. ostreatus* strain 2461, it does not provide the best antiradical scavenging activity compared to the other fungal strains. However, it should be borne in mind that the diversity and effective extract yield of different compounds can vary greatly depending on various factors. Due to this tendency, the antioxidant activity of an extract cannot be attributed to the individual contribution of a particular compound. Because, other significant compounds that were not extracted in this study may have a greater contribution to the antioxidant activity.

Our results emphasize the crucial importance of studying specific strains of cultures, as well as raising awareness of the sustainable use of waste for mushroom cultivation. Some difference in the results of mycelial growth *of P. ostreatus* strains used on solid and liquid nutrient media, antagonistic, antibacterial and radical scavenging activities as well as in total phenolic content of this investigation and compare to literature data of other researchers may be due to the use of different *P. ostreatus* strains, different methods of media preparing, extraction solvents or different assayed microorganisms, and expressed results in other measurement units.

## Conclusion

Two strategies have been used in the present study to increase *P. ostreatus* mycelium production, including the addition of synthetic plant growth regulators such as Ivin, Methyur and Kamethur, and the use of natural waste as a nutrient medium. The article has been updated some new knowledge on the characteristics of *P. ostreatus* strains and their ability to adapt and develop on various nutrient solid media and liquid natural wastes (amaranth flour cake and wheat germ, wheat bran, broken vermicelli and pasta production waste—crumbs). The results indicate the undoubted relevance and prospects of using such wastes in culture media both as monobases and in their combination. It was confirmed that the growth, antibacterial, antiradical scavenging activity as well as total phenolic content were dependent on studied strain. Only the assessment of fungal competition showed that their interaction did not depend on the strain of *P. ostreatus* used in co-cultivation. Further investigations of the compounds responsible for the biological activity of the *P. ostreatus* strains mycelium cultured using the studied wastes would be useful.

## Data Availability

No datasets were generated or analysed during the current study.
